# Modelling and Simulation of MuCell^®^: The Effect of Key Processing Parameters on Cell Size and Weight Reduction

**DOI:** 10.3390/polym14194215

**Published:** 2022-10-08

**Authors:** Yifei Ding, Cian Vyas, Otto Bakker, Srichand Hinduja, Paulo Bartolo

**Affiliations:** 1 Department of Mechanical, Aerospace and Civil Engineering, The University of Manchester, Manchester M13 9PL, UK; 2 Singapore Centre for 3D Printing, School of Mechanical and Aerospace Engineering, Nanyang Technological University, Singapore 637551, Singapore

**Keywords:** cell morphology, MuCell^®^, Moldex 3D, process parameters, simulation, weight reduction

## Abstract

Microcellular injection moulding is an important injection moulding technique to create foaming plastic parts. However, there are no consistent conclusions on the impact of processing parameters on the cell morphology of microcellular injection moulded parts. This paper investigates the influence of the main processing parameters, such as melt temperature, mould temperature, injection pressure, flow rate, shot volume and gas dosage amount, on the average cell size and weight reduction of a talc-reinforced polypropylene square part (165 mm × 165 mm × 3.2 mm), using the commercial software Moldex 3D. The effect of each parameter is investigated considering a range of values and the simulation results were compared with published experimental results. The differences between numerical and experimental trends are discussed.

## 1. Introduction

**Figure 1:**
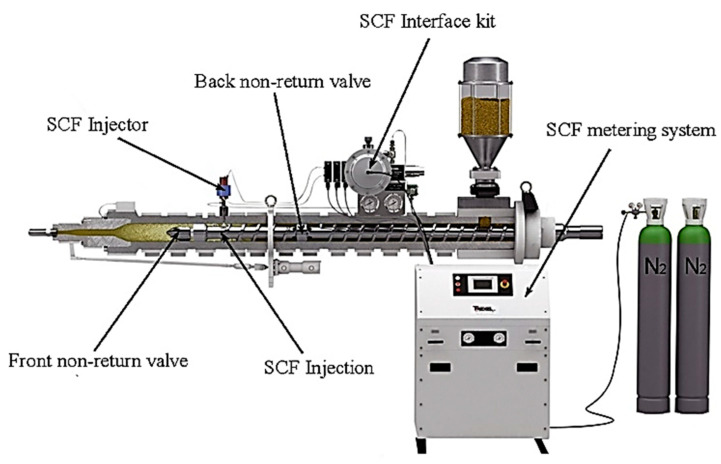
MuCell^®^ machine system [14].

Microcellular injection moulding (MuCell^®^) has drawn significant industrial attention, as it allows for the production of plastic parts with large weight reduction [1,2]. Applications include foaming parts for automotive, aerospace and medical applications among other sectors [3,4]. During the MuCell^®^ process, melted polymer and supercritical fluid (SCF) are mixed under high-pressure conditions in a barrel before the filling stage. During the filling process, a large number of bubbles is formed in the mould cavity as a consequence of the significant pressure drop, which contributes to the formation of a sandwich-like structure (parts with solid skins and foamed core) [5]. Figure 1 presents the MuCell^®^ machine system, including the SCF metering system, SCF interface kit, SCF injector and front and back non-return valve.

However, microcellular injection moulding presents some limitations, such as silver marks on the part surface, caused by some bubbles trapped between the mould cavity and the melt and poor mechanical properties, which limit the wider use of this technology. Two approaches have been proposed to prevent the silver mark formation. The first is to increase the mould temperature in the filling stage to mix the trapped bubbles with the melt. Techniques, such as the rapid mould heating and cooling (RMHC) system [6], dynamic mould temperature control system [7,8], electromagnetic induction heating technology [9] and mould surface coating [10,11,12], were developed based on this principle. The second approach is to stop bubbles from getting trapped between the mould cavity and melt in the filling stage. Techniques, such as gas counter pressure (GCP) system [13,14,15,16,17,18], gas-assisted microcellular injection moulding (GAMIM) [19] and pressure-temperature (P-T) control system [20,21], were developed based on this principle by increasing the mould cavity pressure to a certain level at the beginning of the filling stage. It was also reported that both GCP technology [22] and GAMIM [19] can improve mechanical properties by increasing solid skin thickness. Though all these methods can effectively improve part of the surface and mechanical properties, they require additional equipment to assist, which increases manufacturing costs.

Generally, both silver mark formation and mechanical properties are related to the cell morphology on the microcellular injection moulded parts. Gómez et al. [23] found that the surface roughness can be influenced by changing injection moulding parameters, such as mould temperature, injection velocity, shot volume and gas content. Similar observations were reported by other authors [6,9,24,25,26,27,28,29,30]. Results showed that the surface quality of injected parts increases by increasing both the mould temperature and the injection velocity, while decreasing the mould temperature, the injection velocity and the shot volume or increasing the gas content have a negative impact on the surface quality [20,22,31]. However, Kastner et al. [31] reported that the increase in gas content decreases the average cell size. Moreover, Gómez-Monterde et al. [32] found that the influence of the shot volume on the morphological properties of injected parts, such as skin thickness, cell density and cell size, is not significant. Li et al. [33] found that the increase in the shot size resulted in weight reduction, a decrease in the size and an increase in cell density. These authors also observed that both cell size and cell density increase by increasing the injection velocity [33]. A non-linear impact of mould temperature and cell size was also reported, with results indicating that by increasing the mould temperature, the cell size initially decreases and then increases [33]. Contrarily, Lohr et al. [34] found that the influence of the injection velocity can be neglected. Liu et al. [35] concluded that the cell density decreases in a linear way by decreasing the shot volume and that the effect of gas content on the cell density can be neglected. Mi et al. [36] investigated the influence of several process parameters, such as mould temperature, gas content, injection velocity and shot volume, on cell morphology. They found that, among all considered parameters, the gas content was the most important one regarding the impact on both cell size and cell density and that both mould temperature and injection velocity had a significant influence on skin thickness and cell density. The same trend was also reported by Yoon et al. [37]. Volpe et al. [38] found that the injection temperature decreases the cell size but increases the cell density, while Yuan et al. [39] found that the shot size has a higher impact on both cell size and cell density than the melt temperature, the gas content and the injection velocity. These different studies reveal that there are no consistent conclusions regarding the influence of these parameters on cell morphology [40].

To address this limitation, a systematic simulation study is conducted in this paper to investigate how simulation parameters influence cell morphology and weight reduction in MuCell^®^ parts. A simple model, designed for future experimental validation, and the corresponding cooling system were numerically modelled using the software Moldex 3D. The relationship between key MuCell^®^ process parameters, such as melt temperature, mould temperature, flow rate, injection pressure, shot volume and gas dosage amount, with cell morphology and weight reduction are studied. The obtained relationships are compared with reported experimental results and their differences are analysed. Rather than developing a novel finite element simulation tool, the authors decided to use Moldex 3D as it is a well-accepted software by industry.

## 2. MuCell^®^ Simulation

### 2.1. Part Design and Materials

**Figure 2:**
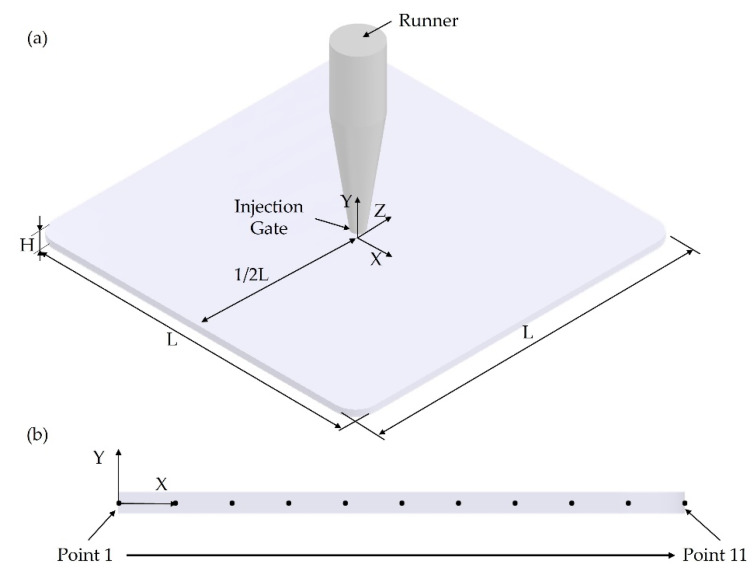
(**a**) The model dimensions and injection gate location and (**b**) selected cross-section area and points considered for analysis. The distance between two consecutive points is 8.25 mm.

The plastic part considered for simulation purposes is presented in Figure 2a. The dimensions of the model are 165 mm × 165 mm × 3.2 mm (L×L×H). The injection gate is a pin gate located at the centre of the part. A high-performance talc-reinforced polypropylene, Sabic PP (PHC27), commonly used for automotive applications, was selected for the simulations. This material presents a melt flow rate (MFR) of 14 g/10 min at 230 °C and 2.16 kg (ISO 1133) and a density of 0.905 g/cm^3^ (ISO 1133). To investigate, in more detail, cell morphology inside the part from the injection gate along the horizontal flow direction, a selected region highlighted in Figure 2a was selected and its cross-section is presented in Figure 2b. Eleven points in the middle of the cross-section were considered for simulation analysis and the distance between them was kept constant.

### 2.2. Cooling System

**Figure 3:**
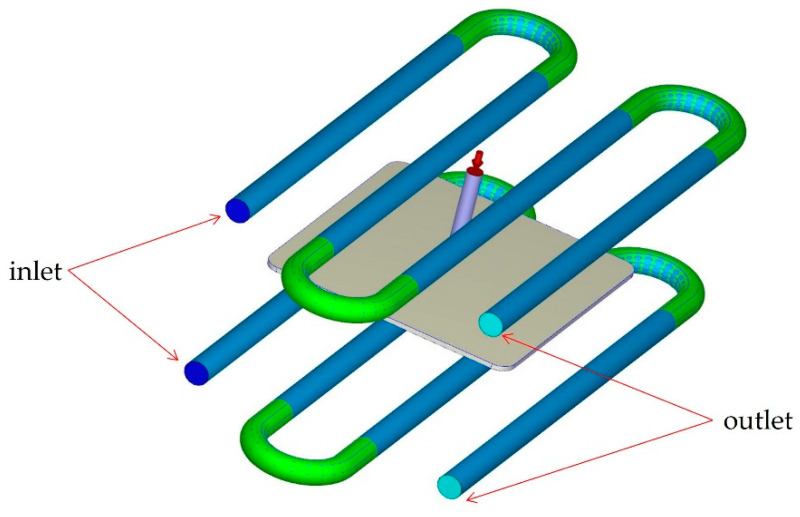
The cooling channels.

The cooling channel layout is presented in Figure 3. It consists of four cooling channels with a diameter of 16 mm, which are located 49.68 mm above and under the part. The distance between the cooling channels is 57 mm. The coolant is water (density: 1.0 g/cm^3^, thermal conductivity: 5.9 × 10^4^ erg/s·cm·K °C, heat capacity: 4.1 × 10^7^ erg/g·K °C) and both the inlet and outlet of the coolant are indicated in Figure 3 as dark-blue and light-blue colours, respectively. The mould material is P20 steel with 7.75 g/cm^3^ of density, heat capacity of 4.62 × 10^6^ erg/g·K and thermal conductivity of 2.9 × 10^6^ erg/s·cm·K.

### 2.3. Processing Conditions and Nucleation Model

Simulations were conducted to investigate the effect of different processing parameters (melting temperature, mould temperature, flow rate, injection pressure, shot volume and gas dosage amount) on both the morphology (cell size) and part’s weight. For each processing parameter, three different values were considered, as shown in Table 1. The middle values in Table 1 are considered as the reference values for the selected material, machine and part geometry (default values).

A commercial plastic injection moulding simulation software, Moldex 3D (CoreTech System Co., Ltd., Zhubei City, Taiwan), was used to simulate the MuCell^®^ process. The mesh type used was the 3-layer Boundary Layer Mesh (BLM), which will generate a three-layer prism mesh from the part surface and fill in the rest of the area with a tetrahedral mesh. The machine selected was the ENGEL-DUO 16050/1700-120, with 120 mm screw diameter. The heat transfer coefficient between the mould base and the melt was automatically calculated by the software solver based on the process conditions. Nitrogen, with molecular weight of 28 g/mol, solubility of 4 × 10^−11^ mol/cm^3^Pa and diffusion coefficient of 8.07 × 10^−5^ cm^2^/s, was selected as it allows for high foaming levels [41].

There are five bubble growth models to simulate the MuCell^®^ process: the Han and Yoo, the Payvar, the Shafi, the Rosner and the modified Han and Yoo models. The Han and Yoo model describes the mass transfer at the interface of the gas bubble as follows [42]:

(1) $$\frac{dP_{D}}{dt}=\frac{1}{R^{2}}\frac{6D\left(R_{g}T\right)\left(\bar{c}-c_{R}\right)}{-1+{\left\{1+\frac{2/R^{3}}{R_{g}T}\left(\frac{P_{D}R^{3}-P_{D0}R_{0}^{3}}{\bar{c}-c_{R}}\right)\right\}}^{1/2}}-\frac{3P_{D}}{R}\frac{dR}{dt}$$

where $P_{D}$ is the bubble pressure, t is the injection time, R is the bubble radius, $P_{D0}$ is the saturation pressure, T is the temperature, D is the diffusion coefficient, $c_{R}$ is the dissolved gas concentration at the bubble surface, $\bar{c}$ is the average dissolved gas concentration and $R_{g}$ is the gas constant.

According to the Payvar model, the bubble growth is described as follows [43]:

(2) $$\frac{dP_{D}}{dt}=\frac{3D\left(R_{g}T\right)\left(\bar{c}-c_{R}\right)}{R^{2}}\left[1+\frac{1}{-1+{\left\{1+\frac{1/R^{3}}{R_{g}T}\left(\frac{P_{D}R^{3}-P_{D0}R_{0}^{3}}{\bar{c}-c_{R}}\right)\right\}}^{\frac{1}{2}}}\right]-\frac{3P_{D}}{R}\frac{dR}{dt}$$

Shafi et al. described the bubble growth according to the following equation [44]:

(3) $$\frac{dP_{D}}{dt}=\frac{36}{5}\frac{R{\left(R_{g}T\right)}^{2}{\left(\bar{c}-c_{R}\right)}^{2}}{P_{D}R^{3}-P_{D0}R_{0}^{3}}-\frac{3P_{D}}{R}\frac{dR}{dt}$$

Rosner et al. described the bubble growth and the shrinkage process by using a sgn function [45]:

(4) $$\frac{dP_{D}}{dt}=sgn\left(\bar{c}-c_{R}\right)\frac{6R{\left(R_{g}T\right)}^{2}{\left(\bar{c}-c_{R}\right)}^{2}}{P_{D}R^{3}-P_{D0}R_{0}^{3}}-\frac{3P_{D}}{R}\frac{dR}{dt}$$

The modified Han and Yoo model also takes into consideration the bubble shrinkage as follows:

(5) $$\frac{dP_{D}}{dt}=\frac{1}{R^{2}}\frac{6D\left(R_{g}T\right)\left(\bar{c}-c_{R}\right)}{-1+{\left\{1+\frac{2/R^{3}}{R_{g}T}\left(\frac{P_{D}R^{3}-P_{D0}R_{0}^{3}}{sgn\left(\bar{c}-c_{R}\right)\left(\bar{c}-c_{R}\right)}\right)\right\}}^{1/2}}-\frac{3P_{D}}{R}\frac{dR}{dt}$$

Based on previous studies, the Payvar’s model corresponds to the fastest bubble growth model, while the Shafi’s model corresponds to the slowest [46]. Both the Rosner and modified Han and Yoon models consider high in-mould pressure conditions, which will lead to bubble shrinkage with different convergence [46,47]. In this study, simulations were conducted based on the dynamic bubble growth model proposed by Han and Yoo, as it was reported to enable good consistency between simulation and experimental results [48,49].

### 2.4. Mathematical Models for MuCell^®^ Simulation

The flow field can be modelled by considering the mass, momentum and energy balance equations as follows [49,50]:

(6) $$\frac{\partial \rho }{\partial t}+\nabla \cdot \rho u=0$$

(7) $$\frac{\partial }{\partial t}\left(\rho u\right)+\nabla \cdot \left(\rho uu+pI-ɳ\left(\nabla u+\nabla u^{T}\right)\right)=\rho g$$

(8) $$\rho C_{P}\left(\frac{\partial T}{\partial t}+u\cdot \nabla T\right)=\nabla \left(k\nabla T\right)+ɳ{\dot{\gamma }}^{2}$$

where ρ is the density of the polymer, t is the injection time, u is the velocity vector, ɳ is the viscosity, p is the pressure, T is the temperature, $C_{P}$ is the specific heat, I is the unit tensor, g is the gravity, k is thermal conductivity tensor and $\dot{\gamma }$ is the shear rate.

In the case of bubble nucleation and growth process, the 3D numerical simulation is applied to describe the dynamic behaviour of the bubble growth, which is coupled with macroscopic molten polymer flow. The radius of bubble growth is given by the following equation [48,51]:

(9) $$\frac{dR}{dt}=\frac{R}{4ɳ}\left(P_{D}-P_{C}-\frac{2\gamma }{R}\right)$$

where R is the bubble radius, $P_{D}$ is the bubble pressure, $P_{C}$ is the ambient pressure and γ is the surface tension.

A thin boundary layer condition was assumed and the dissolved gas concentration profile along the radial direction of a thin shell is described by the following diffusion equation [48]:

(10) $$\frac{\partial c}{\partial t}=D\left[\frac{1}{r^{2}}\frac{\partial }{\partial r}\left(r^{2}\frac{\partial c}{\partial r}\right)\right]$$

where c is the dissolved gas concentration and D is the diffusion coefficient.

Bubble nucleation occurs because the flowing pressure of the molten polymer decreases from the sprue to the mould cavity during the filling process. The cell nucleation rate is expressed through an exponential function of the concentration (mass conservation) of dissolved gas, as follows [48]:

(11) $$J\left(t\right)=f_{0}{\left(\frac{2\gamma }{\pi M_{W}/N_{A}}\right)}^{1/2}exp\left[-\frac{16\pi \gamma^{3}F}{3k_{B}T{\left(\frac{\bar{c}\left(t\right)}{k_{H}}-P_{C}\left(t\right)\right)}^{2}}\right]N_{A}\bar{c}\left(t\right)$$

where $f_{0}$ and F are fitting parameters in the bubble nucleation rate equation, being, respectively, 7.4 × 10^−25^ and 1 × 10^−3^ (default values in Moldex 3D). In this equation $N_{A}$ is the Avogadro number, $k_{B}$ is the Boltzmann constant, $k_{H}$ is the solubility parameter and $M_{W}$ is the gas molecular weight. The threshold of bubble nucleation rate is set as 0.1 cm^−3 ^s^−1^, which is the default value in Moldex 3D.

The average concentration of super critical fluid (SCF) dissolved in the polymer at a time t is given by [48]:

(12) $$\bar{c}\left(t\right)V_{L0}=c_{0}V_{L0}-\int_{0}^{t}\frac{4\pi }{3}R^{3}\left(t-t^{\prime },t^{\prime }\right)\frac{P_{D}\left(t-t^{\prime },t^{\prime }\right)}{R_{g}T}J\left(t^{\prime }\right)V_{L0}dt^{\prime }$$

where $V_{L0}$ is the volume of the polymer.

The viscosity of the polymer melt is influenced by the SCF dissolved in the polymer melt. Thus, the modified cross model with Arrhenius temperature dependence was considered to describe the viscosity [46]:

(13) $${ɳ}_{p}\left(T,\dot{\gamma }\right)=\frac{{ɳ}_{0}\left(T\right)}{1+{\left({ɳ}_{0}\dot{\gamma }/\tau^{*}\right)}^{1-n}}$$

(14) $${ɳ}_{0}\left(T\right)=Bexp\left(\frac{T_{b}}{T}\right)$$

where n is the power law index, which was set as 1 in the simulation, ${ɳ}_{0}$ is the zero-shear viscosity, $\tau^{*}$ is a parameter that describes the transition region between zero shear rate and the power law region of the viscosity curve and B is a pre-exponential factor. For simplicity, the molten material was assumed to have a Newtonian behaviour. The accuracy of this assumption was previously demonstrated by Taki [48] and Xi et al. [52].

## 3. Simulation Results

### 3.1. Reference Case

**Figure 4:**
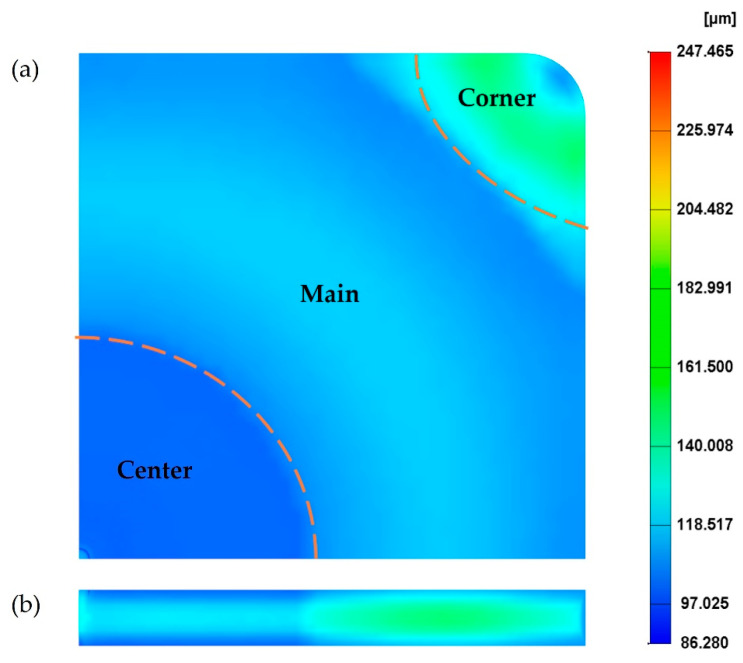
The cell distribution at the surface (**a**) and cross-section (**b**) of the MuCell^®^ part considering the reference processing conditions.

For the reference case (see processing conditions in Table 1), the cell size on the MuCell^®^ part surface is presented in Figure 4a. As the results at the top and bottom surface are the same, only the results on the top surface are presented. From Figure 4a, it is possible to observe that the cell distribution can be divided into three main areas around the injection gate: centre, main and corner. At the centre, the cell size is around 100 µm, which corresponds to the smallest cell size on the part surface. In the main area, cell sizes range from 100 µm to 130 µm. Finally, the largest cells are located at the corner areas, far from the injection gate, with sizes ranging from 130 µm to 180 µm. These differences are due to the cell formation process in those areas, which occurs at different stages in the injection moulding cycle [5]. The cells at the main and corner areas start forming at the filling stage, while the cells at the centre area only start forming at the cooling stage due to high pressure. Overall, the average cell size is 129.4 µm and the weight reduction of the part, in comparison to a solid one, is 25.5%.

**Figure 5:**
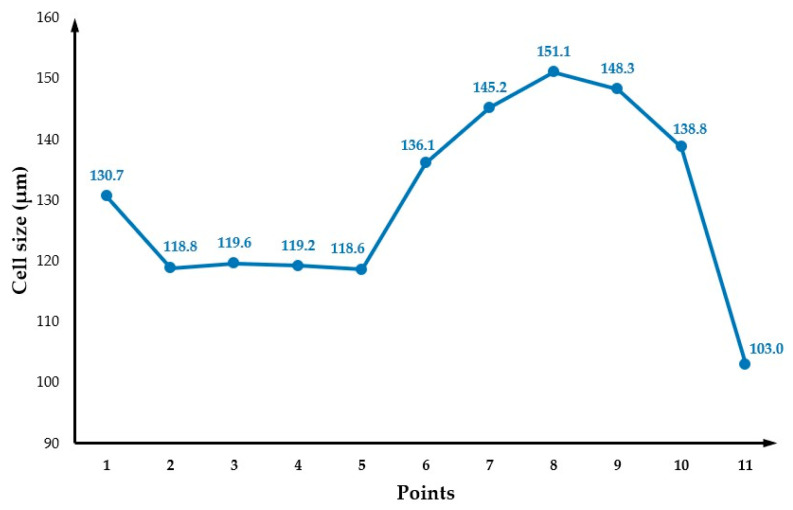
The cell size variation across the cross-section.

Figure 4b presents the cell distribution at the cross-section. As observed, the cross-section exhibits a typical sandwich structure, with unfoamed (small cells) skin and foamed core. Moreover, cells far from the injection gate tend to be larger. Values of cell size at different points across the cross-section (Figure 2b) are presented in Figure 5. Similar to what was observed for the part’s surface, a gradient of pore sizes is visible and two main regions can be identified. Points from 1 to 5 (centre area) show the smallest cell sizes (130 µm at point 1). Points from 6 to 10 (main area) show the largest cell sizes (151 µm at point 8). From point 8, cell size starts to slightly decrease as those points are approaching the mould surface. Finally, a significant reduction in pore size was observed at point 11 (103 µm), which is located at the mould surface.

### 3.2. The Effect of Changing the Mould Temperature

**Figure 6:**
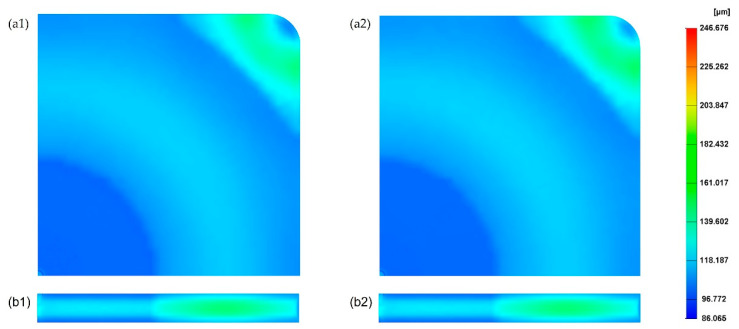
Cell size distribution on the surface and cross-section of the MuCell^®^ part considering mould temperature of 20 °C (**a1**,**b1**) and 60 °C (**a2**,**b2**).

**Figure 7:**
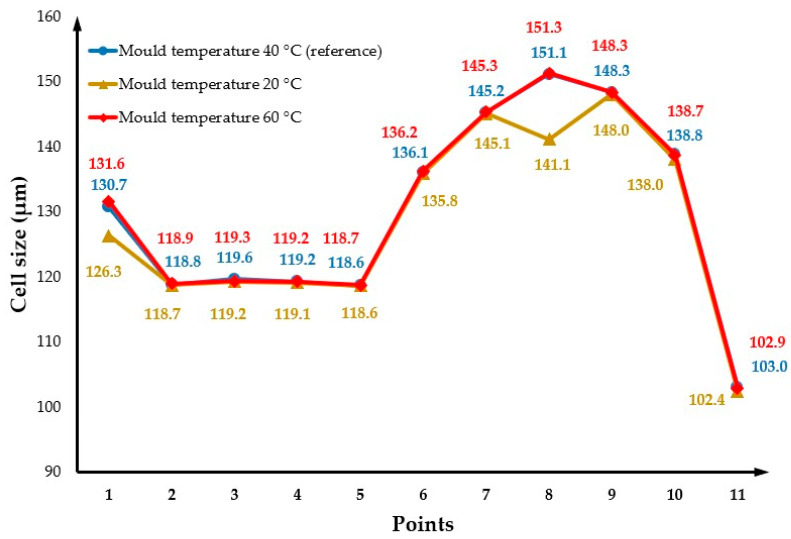
Cross-section variation in cell size as a function of mould temperature.

**Figure 8:**
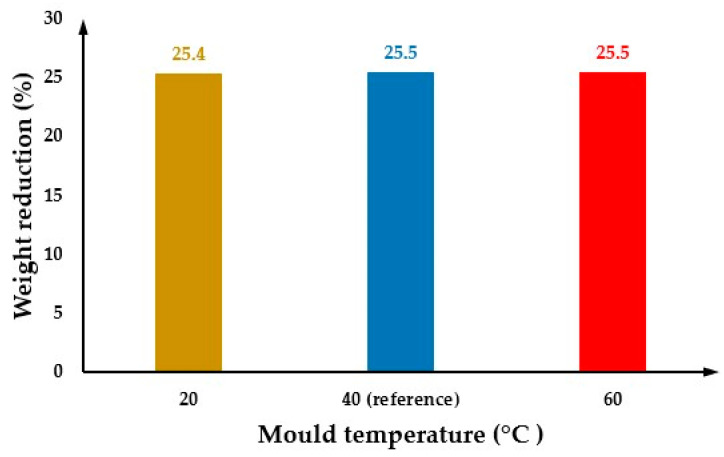
The weight reduction as a function of mould temperature.

Figure 6 shows the cell size values at both the part surface and cross-section considering the two different mould temperatures (20 °C and 60 °C), lower and higher than the reference. All the other parameters were kept constant assuming the reference values. By comparing these results with the reference case (Figure 4), it is possible to observe that the effect of changing the mould temperature on cell size can be neglected. The cell size average is 128.9 µm for a mould temperature of 20 °C and 129.3 µm for a mould temperature of 60 °C. Figure 7 presents the cross-section variation in the cell size for different mould temperatures. Results show no significant differences in cell sizes as a function of mould temperature, with the results obtained for 60 °C being quite close to those obtained for the reference conditions. However, for a mould temperature of 20 °C, the cell size values near the injection gate (point 1) and at point 8 (maximum cell size values) are lower than the cell size values at the same positions for both 60 °C and reference conditions. The effect of the mould temperature on the part weight reduction seems also to be neglectable, as shown in Figure 8.

These results require further experimental validation as they are not in agreement with experimental results, suggesting that the mould temperature is a key factor determining the cell morphology of MuCell^®^ parts [6,7,9,20,23,29]. Moreover, it was reported that high mould temperatures result in significant weight reduction [20].

### 3.3. The Effect of Gas Dosage Amount

**Figure 9:**
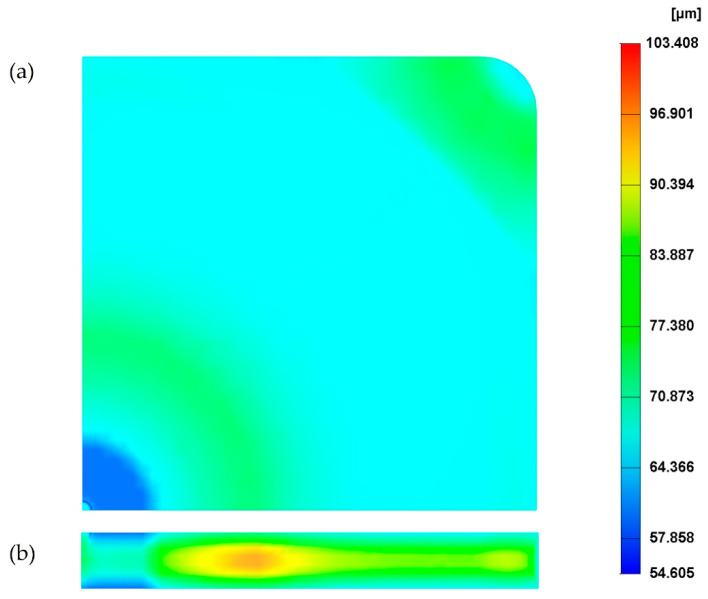
Cell size distribution on the surface (**a**) and cross-section (**b**) of the MuCell^®^ part considering a gas dosage amount is 0.5%.

**Figure 10:**
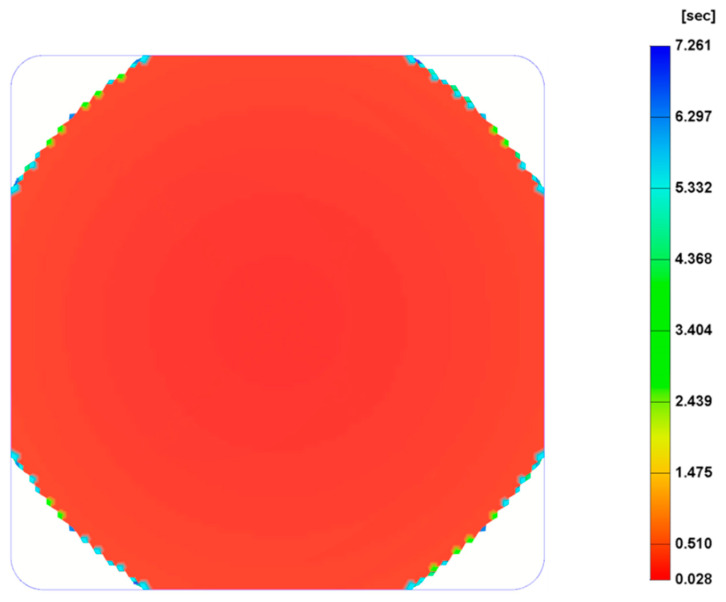
Melt front time when the gas dosage amount is 0.1%.

**Figure 11:**
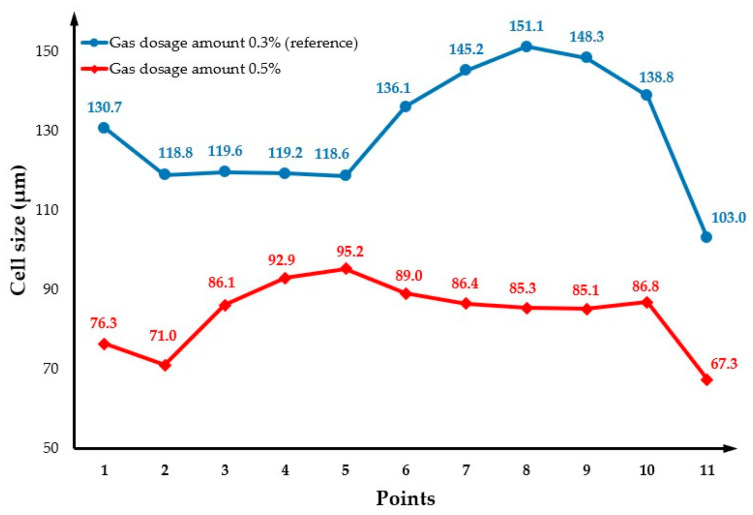
Cross-section variation in cell size as a function of gas dosage amount.

**Figure 12:**
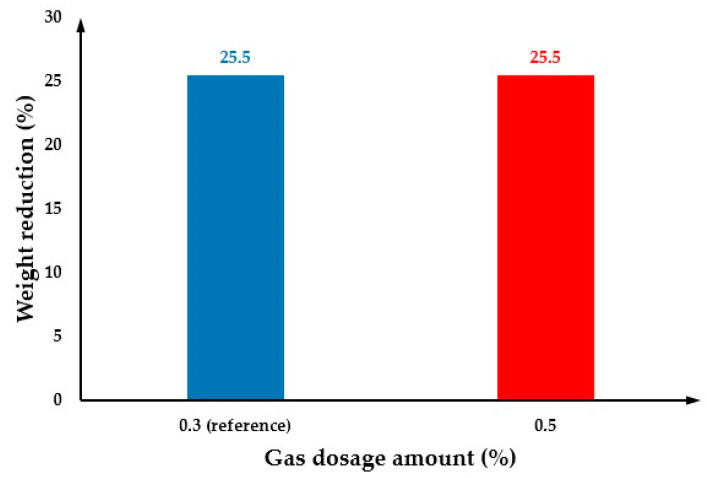
The weight reduction as a function of gas dosage amount.

The effect of three different gas dosage amount values, 0.1%, 0.3% (reference) and 0.5%, on the cell size was investigated and the results are presented in Figure 9, except for the gas dose amount of 0.1%, which is not presented due to the short shot (Figure 10). As observed, the cell size significantly changes by increasing the gas dosage amount from 0.3% to 0.5%. Results show that, for a gas dosage amount of 0.3%, the average cell size at the surface is 140 µm and the overall cell size average is 129.4 µm, while for a gas dosage amount of 0.5% the cell size at the surface is 100 µm and the overall cell size average is 78.9 µm. In comparison to the reference case (Figure 4), the centre area is smaller, while the main area is bigger. This can be explained by the fact that when the amount of gas mixed with the melted polymer increases, the number of bubbles formed during the filling stage also increases. However, as a large number of bubbles grows in a limited area, which contributes to increasing the interactions between bubbles, suppressing the growth of their surrounding bubbles. Moreover, large cells are forming in the main area. Figure 9b shows the cell size variation at the cross-section when the gas dosage amount is 0.5%, while the cell size variation for different gas dosage amounts is presented in Figure 11. In this case, different trends can be observed. Overall, cell size values decrease by increasing the gas dosage amount. For high gas dosage amounts, the largest cell sizes seem to occur in cross-section regions near the injection gate (point 5 for a gas dosage amount of 0.5% and point 8 for a gas dosage amount of 0.3%), while the lowest cell size value occurs at point 2 (near the injection gate). However, despite these morphological differences, the overall weight reduction (25.5%) is the same for the two different gas dosage values, as shown in Figure 12.

### 3.4. The Effect of Changing Melt Temperature

**Figure 13:**
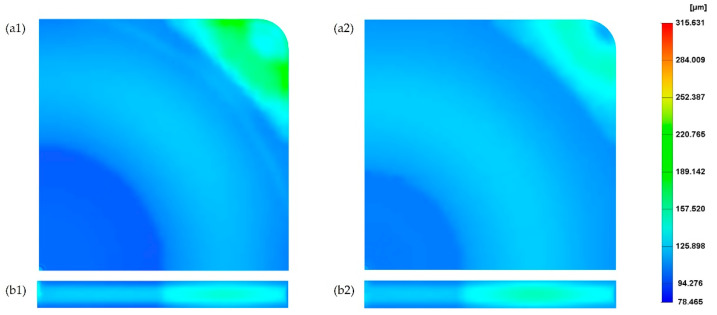
The cell distribution at the surface and cross-section of the MuCell^®^ part considering a melt temperature of 200 °C (**a1**,**b1**) and 240 °C (**a2**,**b2**).

**Figure 14:**
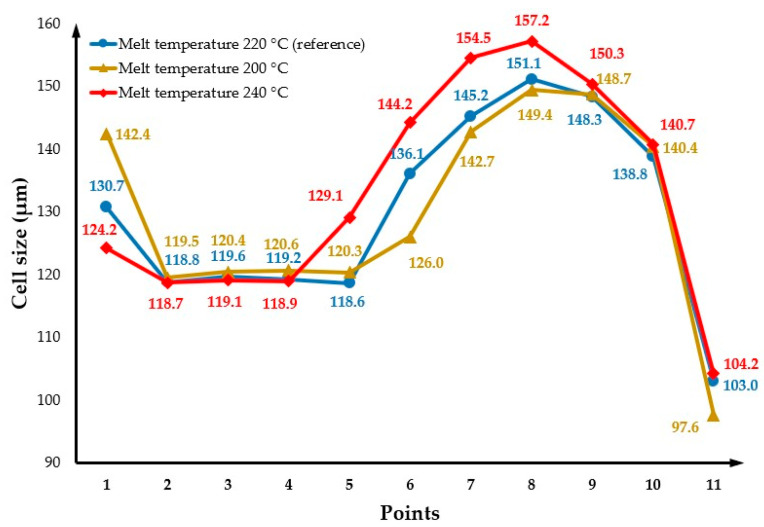
Cross-section variation in cell size as a function of melt temperature.

**Figure 15:**
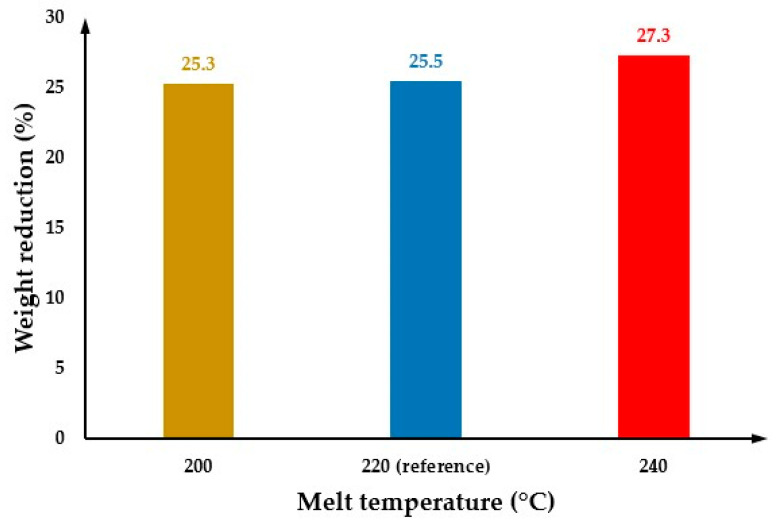
The weight reduction as a function of melt temperature.

The effect of melt temperature on cell morphology was investigated considering three different temperatures (200 °C, 240 °C and the reference melt temperature of 220 °C). Figure 13(a1,b1) and (a1,b2) show the cell distribution on the part surface and cross-section for melt temperatures of 200 °C and 240 °C, respectively. In comparison to the reference condition, it is possible to observe that cell morphology changes can be ignored when the melt temperature decreases to 200 °C, while the average cell size slightly increases to 133.4 µm by increasing the melt temperature to 240 °C. A comparison of the effects of melt temperature on the cell size across the cross-section of the part is presented in Figure 14. As shown, the cell size at points close to the injection gate (points 2 to 4) and the mould wall (points 9 to 11) is the same in both melt temperature conditions. However, for points in the middle of the cavity (points 5 to 8), the cell size increases by increasing the melt temperature, as high melt temperatures provide more time for cell growth. The weight reduction as a function of melt temperature is summarised in Figure 15. As observed, the weight reduction slightly increases when the melt temperature increases to 240 °C. This phenomenon might be associated with the longer cooling time, which provides more time for the bubbles to grow.

### 3.5. The Effect of Changing the Injection Pressure

**Figure 16:**
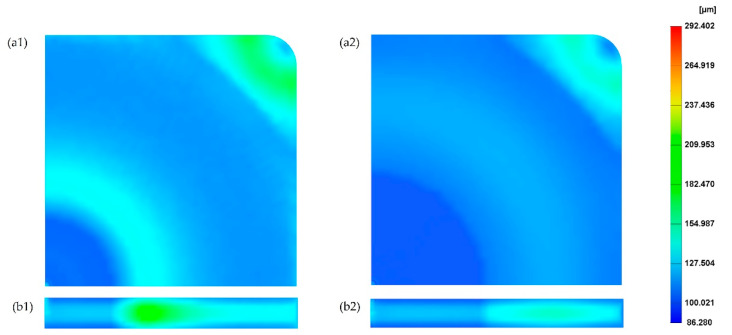
The cell distribution on the part considering an injection pressure of 10 MPa (**a1**,**b1**) and 30 MPa (**a2**,**b2**).

**Figure 17:**
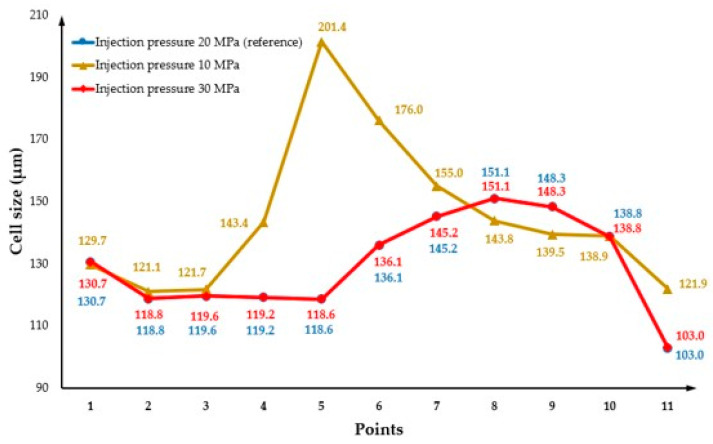
Cross-section variation in cell size as a function of injection pressure.

**Figure 18:**
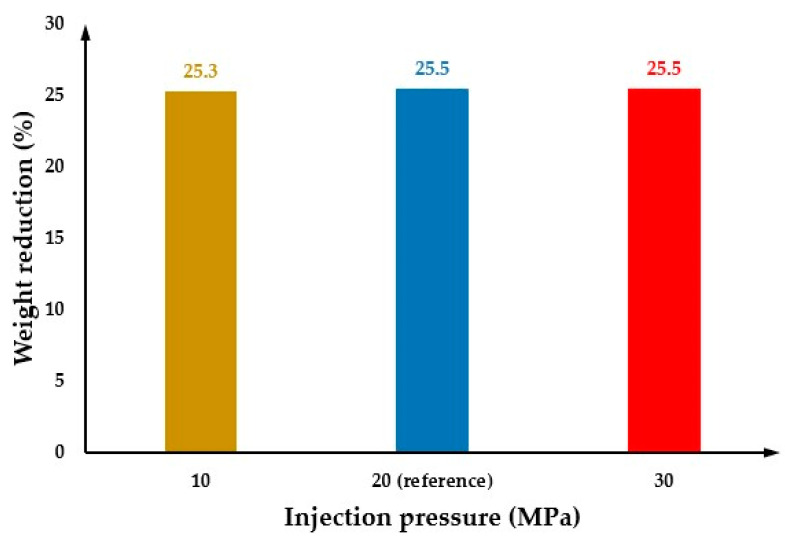
The weight reduction as a function of injection pressure.

The effect of injection pressure on cell morphology was investigated considering two different injection pressures (10 MPa and 30 MPa) and the results were compared with the reference injection pressure (20 MPa). Figure 16(a2) shows the cell morphology at the surface of the MuCell^®^ part for an injection pressure of 30MPa. As observed, the average cell size is equal to the values obtained for the reference injection pressure (129.4 µm). Figure 16(a1,b1) show the cell morphology on both the part surface and the cross-section for a lower injection pressure (10 MPa). In this case, results show a region between the main and centre areas of the mould, where the cell size is significantly higher. This can also be observed in Figure 17, describing the cell size variation for different injection pressures along the cross-section of the plastic part. As observed, for low injection pressure, the cell size significantly increases to 201.4 µm from point 3 to 5 and then decreases to 138.9 µm at point 10. At low injection pressures, the bubbles near the injection gate can start growing earlier and keep growing during both the filling and cooling stages. This contributes to the formation of large cells near the injection gate. However, it was not possible to observe any significant effect of the injection pressure on the weight reduction, as shown in Figure 18.

### 3.6. The Effect of Changing the Flow Rate

**Figure 19:**
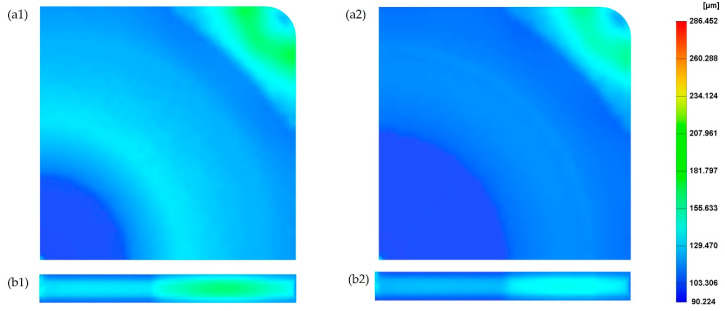
The cell distribution at the surface and cross-section of the MuCell^®^ part considering a flow rate of 295.5 cm^3^/s (**a1**,**b1**) and 689.5 cm^3^/s (**a2**,**b2**).

**Figure 20:**
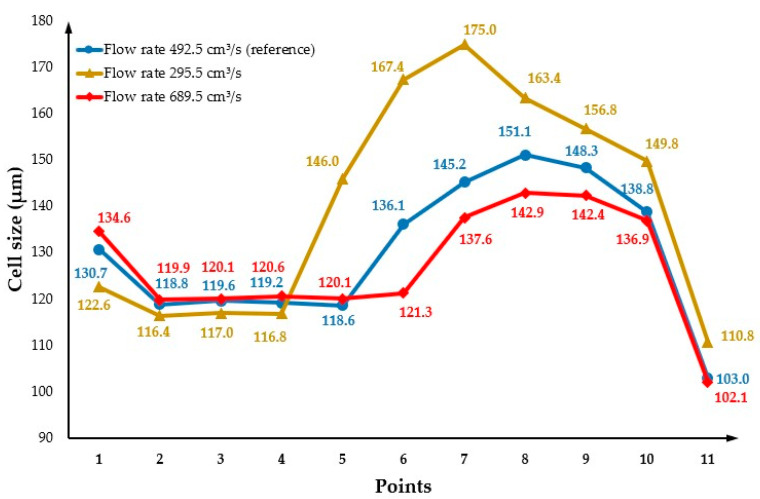
Cross-section variation in cell size as a function of flow rate.

**Figure 21:**
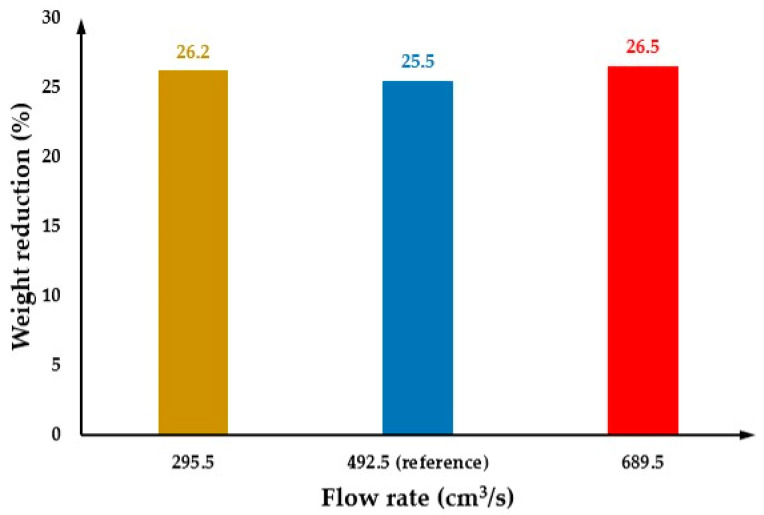
The weight reduction as a function of flow rate.

The effect of flow rate on cell morphology was investigated considering the following flow rates: 295.5 cm^3^/s and 689.5 cm^3^/s. Results were also compared with the reference flow rate of 492.5 cm^3^/s. Figure 19(a1, b1) and (a2, b2) show the cell morphology of the MuCell^®^ part (surface and cross-section) considering the flow rates of 295.5 cm^3^/s and 689.5 cm^3^/s, respectively. As observed, the average cell size slightly increases (142.1 µm) by decreasing the flow rate to 295.5 cm^3^/s concerning the reference conditions. However, by increasing the flow rate up to 689.5 cm^3^/s, the average cell size decreases to 127.0 µm. This trend can be also observed in Figure 20. Moreover, the centre area becomes larger by increasing the flow rate. Generally, the effect of flow rate on cell morphology is the same as the injection pressure, as both parameters are related to the pressure. This can be observed by comparing Figure 17 and Figure 20. However, as indicated in Figure 21, results show no significant impact of the flow rate on weight reduction.

### 3.7. The Effect of Changing the Shot Volume

**Figure 22:**
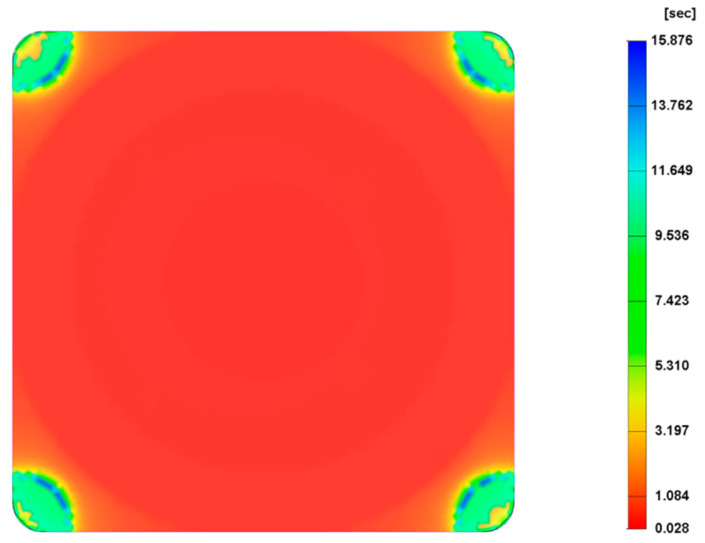
Melt front time for a shot volume of 85%.

**Figure 23:**
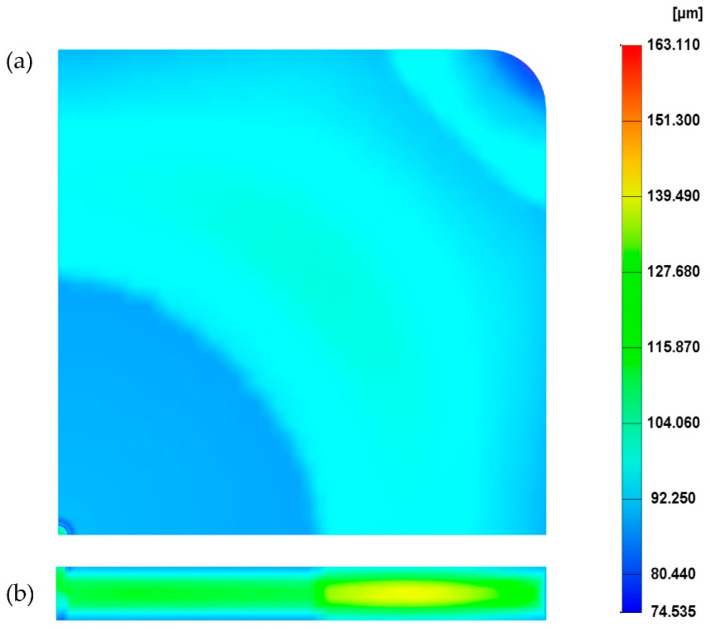
The cell distribution at the surface (**a**) and cross-section (**b**) of the MuCell^®^ part considering a shot volume of 95%.

**Figure 24:**
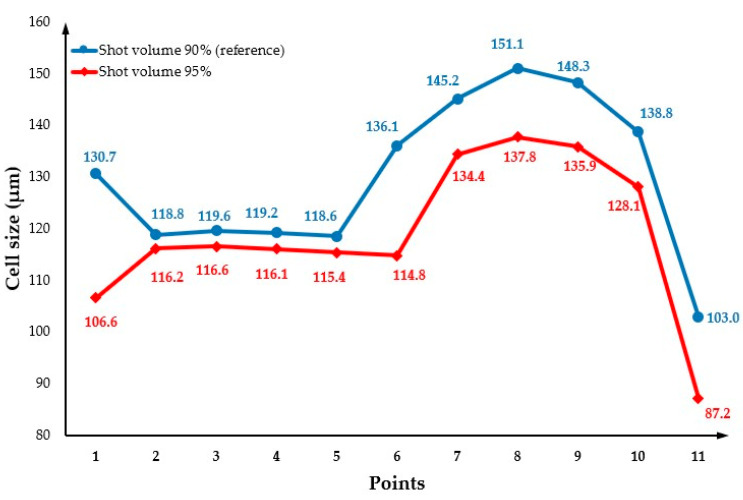
Cross-section variation in cell size as a function of shot volume.

**Figure 25:**
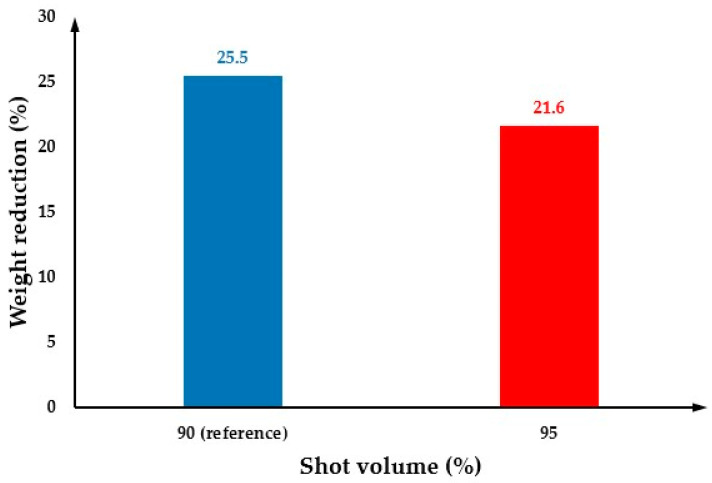
The weight reduction as a function of shot volume.

The effect of the shot volume on cell morphology was investigated considering shot volumes of 85% and 95% and the results were compared with the reference shot volume of 90%. When the shot volume is 85%, the short shot occurred (Figure 22), so the relevant results are not presented. Figure 23 shows the cell morphology of the MuCell^®^ part at both the surface and cross-section for a shot volume of 95%. As observed in both Figure 23 and Figure 24, the cell size significantly decreases (115.6 µm) in comparison to the reference conditions (129.4 µm), because more material is injected into the mould cavity, making difficult the bubble growth process due to a packing effect. Moreover, the weight reduction also decreases, as shown in Figure 25, indicating that the weight reduction can be directly controlled by adjusting the amount of shot volume.

## 4. Conclusions and Future Work

An extensive simulation experiment investigating the effect of processing conditions on cell morphology and weight reduction in a MuCell^®^ part was presented and discussed. A summary of the obtained results is presented in Table 2, together with published experimental results for comparison.

As observed from the simulation results, the gas dosage amount and shot volume are the most important factors determining cell morphology. Simulation results show that by increasing the gas dosage amount, the cell size considerably decreases and this trend is aligned with published experimental results [23,35,36], except for some results reported for polyetherimide (PEI) [33]. This difference is due to the use of high mould temperature to cause cell coalescence as they grow. Similar results were also obtained by increasing the shot volume and the results were also in agreement with experimental results [5,33,35,39], except in one reported case based on the use of acrylonitrile butadiene styrene (ABS) [23]. However, this difference can be attributed to the high SCF content considered in that study that causes cell coalescence. The injection pressure and flow rate also have an impact, but less significant on the cell morphology than gas dosage and shot volume. Both injection pressure and flow rate influence the cell morphology by changing the cavity pressure. The decrease in both injection pressure and flow rate decreases the pressure cavity during the filling stage, providing more time for cells to grow. These observations are also aligned with previously reported experiments [23,33].

The melt temperature is, among the considered parameters, the least impactful on cell morphology. As shown, the cell size slightly increases by increasing the temperature. A similar trend was reported for experiments using thermoplastic polyurethane (TPU) [36], while researchers who investigated polyamide 66 and glass fibre (PA66/GF) obtained the opposite result [38]. Finally, the mould temperature seems to have a neglectable effect on cell morphology. However, this is not aligned with what was previously reported based on experimental results [20,22,23], which can be attributed to the narrow range of temperatures considered in this research.

Regarding the weight reduction, simulation results show that the shot volume is an impactful parameter, with the weight reduction increasing by decreasing the shot volume. These observations are aligned with previously reported experimental results [23,33,42]. However, all the other parameters show a neglectable effect on the weight reduction, a result that is not aligned with previously reported experimental results [33,38]. These differences might be caused by material differences and the corresponding impact on the material rheological behaviour.

In the future, the simulation conditions will be experimentally tested to understand if the reasons for some of the differences in terms of the trends of the impact of the different processing parameters on both cell size and weight reduction are related to material differences or related to the range of values considered in this study. Such experimental results will also allow us to understand how reasonable the assumption was of considering the molten material as a Newtonian fluid, which can be an eventual limitation of the software.

## Figures and Tables

**Table 1:** Processing conditions.

Processing Parameters	Value Range		
	Low	Middle (Reference)	High
Melt temperature (°C)	200	220	240
Mould temperature (°C)	20	40	60
Flow rate (cm^3^/s)	295.5 (30%)	492.5 (50%)	689.5 (70%)
Injection pressure (MPa)	10	20	30
Shot volume (%)	85	90	95
Gas dosage amount (%)	0.1	0.3	0.5

**Table 2:** The summary of simulation results and published experiment results (PPS: poly (phenylene sulfide), PA6: polyamide-6, PS: polystyrene).

Name	Material	Parameters, Their Changes and Corresponding Results																		
		Mould Temperature			Gas Dosage Amount		Melt Temperature				Injection Pressure				Flow Rate				Shot Volume	
		Changes		Average Cell Size	Changes	Average Cell Size	Changes		Average Cell Size		Changes		Average Cell Size		Changes		Average Cell Size		Changes	Average Cells Size
Simulation	PP	↑	↓	no	↑	↓	↑	↓	↑	no	↑	↓	no	↑	↑	↓	↓	↑	↑	↓
Gómez-Monterde et al. [23]	ABS	↑		↓	↑	↓									↑		↓		↓	↓
Kastner et al. [31] and Gómez-Monterde et al. [53]	PP/GF																			
Li et al. [33]	PEI				↑	↑									↑		↓		↑	↓
Liu et al. [35]	PPS				↑	↓													↑	↓
Mi et al. [36]	TPU				↑	↓	↑		↑											
Volpe et al. [38]	PA66/GF						↑		↓											
Yuan et al. [39]	PA6																		↑	↓
Chen et al. [20] and Chen et al. [22]	PS	↑		↑																
